# A Knowledge‐Guided Graph Learning Approach Bridging Phenotype‐ and Target‐Based Drug Discovery

**DOI:** 10.1002/advs.202412402

**Published:** 2025-03-06

**Authors:** Qing Ye, Yundian Zeng, Linlong Jiang, Yu Kang, Peichen Pan, Jiming Chen, Yafeng Deng, Haitao Zhao, Shibo He, Tingjun Hou, Chang‐Yu Hsieh

**Affiliations:** ^1^ College of Control Science and Engineering Zhejiang University Hangzhou Zhejiang 310027 China; ^2^ College of Pharmaceutical Sciences Zhejiang University Hangzhou Zhejiang 310058 China; ^3^ CarbonSilicon AI Technology Co., Ltd Hangzhou Zhejiang 310018 China; ^4^ Center for Intelligent and Biomimetic Systems Shenzhen Institutes of Advanced Technology Chinese Academy of Sciences Shenzhen Guangdong 440305 China

**Keywords:** biological networks, drug target discovery, graph representation learning, phenotypic screening, transcriptomics

## Abstract

Discovering therapeutic molecules requires the integration of both phenotype‐based drug discovery (PDD) and target‐based drug discovery (TDD). However, this integration remains challenging due to the inherent heterogeneity, noise, and bias present in biomedical data. In this study, Knowledge‐Guided Drug Relational Predictor (KGDRP), a graph representation learning approach is developed that effectively integrates multimodal biomedical data, including network data containing biological system information, gene expression data, and sequence data that incorporates chemical molecular structures, all within a heterogeneous graph (HG) structure. By incorporating biomedical HG (BioHG) into a heterogeneous graph neural network (HGNN)‐based architecture, KGDRP exhibits a remarkable 12% improvement compared to previous methods in real‐world screening scenarios. Notably, the biology‐informed representation, derived from KGDRP, significantly enhance target prioritization by 26% in drug target discovery. Furthermore, zero‐shot evaluation on COVID‐19 exhibited a notably higher success rate in identifying diverse potential drugs. The utilization of BioHG facilitates a unique KGDRP‐based analysis of cell‐target‐drug interactions, thereby enabling the elucidation of drug mechanisms. Overall, KGDRP provides a robust infrastructure for the seamlessly integration of multimodal data and biomedical networks, effectively accelerating PDD, guiding therapeutic target discovery, and ultimately expediting therapeutic molecule discovery.

## Graphical abstract

Knowledge‐Guided Drug Relational Predictor (KGDRP), a graph representation learning approach, effectively integrates multiple omics data, including biological network data, gene expression data, and sequence data that incorporates chemical molecular structures. By offering a robust framework for generating biologically meaningful explanations, KGDRP accelerates phenotypic drug discovery, guides therapeutic target identification, and facilitates the discovery of omics‐based therapeutic molecules.

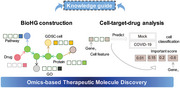

## 1 Introduction

Phenotype‐based drug discovery (PDD) and target‐based drug discovery (TDD) are two major approaches in drug discovery. Historically, the primary method for drug discovery rested upon phenotype‐based techniques, involving the observation of molecule‐induced therapeutic effects on disease phenotypes in cellular models, and animals. The advent of molecular biology revolution in the 1980s shifted the focus to TDD. Since 2011, PDD has resurged due to the unexpected finding that most FDA‐approved first‐in‐class drugs (1999‐2008) were still empirically discovered, without relying on a target hypothesis.^[^ ^1^ ^]^ According to the extensive review of Arash Sadri, among 1144 approved small‐molecule drugs, only 123 of them were discovered by purely target‐based assay methods, and that the rest have to be described as discovered through phenotypic approach.^[^ ^2^ ^]^ Besides, given that the therapeutic effects of many drugs are conducted by complex mechanisms, PDD is particularly crucial for complex diseases with unclear pathophysiology such as cancer.^[^ ^3^ ^]^ However, PDD alone remains inadequate in elucidating the mechanism of actions (MoA) for bioactive compounds. Specifically, the identification of drug targets through PDD retains substantial significance for elucidating disease pathogenesis and advancing drug development.^[^ ^4^ ^]^ Such identification not only provides insights into the MoA but also reveals resistance factors, expanded the scope of “druggable” targets, and thereby reduce the inherent risks of setbacks in drug research and development (R&D).^[^ ^5^ ^]^

From the perspective of PDD and TDD fusion, two tasks in the drug discovery could benefit, drug response prediction and drug target discovery. Notably, the fusion brings mutual benefits. The targets identified through TDD can provide mechanistic explanations for the drug phenotypic effects observed in PDD. By analyzing gene expression profiling that reflect the molecular characteristics of cells, genes crucial to drug treatment can be discovered, thereby inferring potential therapeutic target. To incorporate PDD and TDD into a unified framework, graph‐based approaches could be an effective fusion method at the data level.^[^ ^6^ ^]^ Several studies have developed graph‐based methods that integrate biological knowledge data, such as drug targets, to enrich the representation of both cell lines and drugs.^[^ ^7^ ^]^ For instance, Minwoo Pak et al. introduced NetGP, a module employing network propagation techniques to compute gene perturbation scores. This integration enhances drug response prediction by incorporating drug target data, thereby contributing to the advancement of predictive modeling in pharmacogenomics research.^[^ ^8^ ^]^ While current methods have successfully incorporated biological processes linked to drugs through pathway enrichment, the aspect of drug target discovery has not received sufficient investigation.^[^ ^7^, ^9^ ^]^ As for drug target discovery in the view of fusion of PDD and TDD, this task typically relates with the interpretability of drug response model or incorporating the drug response data in modelling to improve the drug target interaction (DTI) prediction.^[^ ^6^, ^10^ ^]^ These computational approaches tend to focus exclusively on either PDD or TDD due to the data heterogeneity. Another challenge in merging PDD and TDD is that the drugs involved in these two approaches may not overlap, resulting in some drugs lacking connections within the biomedical graph, which is called cold‐start problem. Such limitation could constrain the application of graph‐based computational models in phenotypic drug screening.

In this study, we aim to develop a framework to integrate the PDD and TDD data for both enhancing the drug response prediction and drug target discovery within a larger biomedical network. Due to heterogeneity, unavoidable noise as well as the bias within the biomedical data, computational fusion of PDD and TDD based on heterogeneous biomedical graphs remains challenging. Therefore, a key issue is how to integrate high‐dimensional phenotypic data into heterogeneous networks and enable the model to learn complex biological correlations. Here, we present a multimodal data fusion framework called Knowledge‐Guided Drug Relational Predictor (KGDRP). This framework seamlessly integrates biological network data containing biological system information, gene expression data, and sequence data incorporating chemical molecular structures, within a heterogeneous graph (HG) structure. Through extensive end‐to‐end representation learning on the biology‐informed HG, KGDRP generates comprehensive representations for drugs, proteins, and cell lines. To address the drug cold‐start problem, KGDRP introduces a transformation function for drug nodes, which learns a mapping function from chemical structure to knowledge‐informed drug representations. KGDRP was evaluated for both drug response prediction and drug target prediction, showing comparable predictive performance in drug response against six competitive state‐of‐the‐art methods. Notably, in cold‐start scenarios, KGDRP achieved a substantial 12% increase in Spearman's Correlation Coefficient (SCC), demonstrating significant improvement. The generalization ability of KGDRP was further validated through its transferability assessment from high‐throughput screens to pre‐clinical drug screening. Furthermore, the biology‐informed representation by KGDRP inherently facilitates the elucidation of drug mechanisms of action (MoA), which is fully verified by the evaluation results of target prioritization task. In a zero‐shot evaluation for drug repurposing for COVID‐19, we exhibit the advantages and potential of this computational framework. Overall, the knowledge‐informed framework exhibits the remarkable potential for the refinement of predictive precision in drug phenotype screening, pinpointing of novel therapeutic targets, and amplifies the generalization capacity for clinical predictive performance.

## 2 Results

### 2.1 Graph Deep Learning Paradigm Integrating Biomedical Networks Benefits Phenotypic Screening Prediction

**Figure 1:**
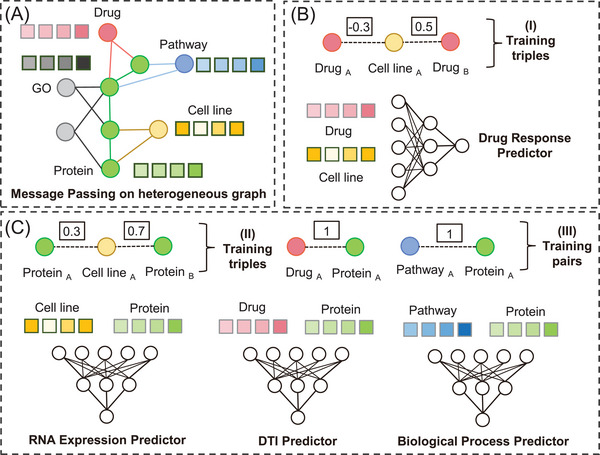
KGDRP framework illustration. A) Heterogeneous message passing on BioHG. The core of the BioHG lies the complex interaction among drugs, proteins, and cell lines. To enable robust representation and model interpretability, we augment the BioHG with gene ontology (GO) and pathway information. This module allows for the updating of node embeddings, each representing a distinct biomedical entity, through the process of heterogeneous message passing. B) Training Samples Generation and predictor training for drug response prediction task. For drug response data, the task is reframed as a response comparison task rather than a value fitting task. Utilizing embeddings derived from the GNN model, we generate triples of the form *drug_A_* − *cell* *line_A_* − *drug_B_*, which are then employed to train the drug response predictor, integrated as a MLP within the KGDRP architecture. C) Three auxiliary predictors are implemented to facilitate effective and robust representation learning. These predictors leverage three types of relational data: RNA expression data of cell lines, DTI, and protein‐pathway associations, integrated to construct foundational training data utilizing the BioHG. Given the inherent susceptibility of RNA expression data to fluctuations influenced by experimental conditions, we redefine the task from fitting values to a more robust expression comparison approach within the KGDRP framework. Numerical annotations on the edges between proteins and cell lines represent expression values, used for RNA expression predictor training, not in the message‐passing process.

For drug response prediction, existing methods have typically treated transcriptional data solely as feature vectors for input into deep learning models. However, this approach presents several challenges in incorporating biological knowledge. First, the integration of diverse biomedical data types is challenging due to their inherent heterogeneity. Second, directly combining multiple‐source datasets can create high‐dimensional feature spaces, potentially leading to overfitting and significantly increase computational costs. Here, our approach introduces a novel technique wherein cell lines are represented as nodes within a BioHG (**Figure** **1**; Table S1, Supporting Information).

#### 2.1.1 Overview of BioHG

In recent years, several large biomedical knowledge graphs have developed to support various drug discovery tasks.^[^ ^11^ ^]^ However, their complexity, unbalanced structures, and inherent noise often limit their ability to improve prediction accuracy or model interpretability.^[^ ^12^ ^]^ Aiming to enhance drug response prediction and prioritize drug targets, our approach focuses on integrating data from PDD and TDD more effectively, while avoiding the inclusion of irrelevant information. BioHG centers on describing the interactions among drugs, proteins, and cell lines, with node and edge selection guided by task relevance and data availability. Consequently, the architecture of BioHG incorporates key data types: drug response data capturing drug‐cell relationships, drug‐target data describing drug‐protein interactions, and RNA expression profiles of cell lines representing protein‐cell line relationships. However, inferring the biological function of proteins, which is the key part of decoding the drug mechanism, is challenging when relying solely on the three relational data sources mentioned above. Benefited from the availability of abundant omics data, BioHG incorporates protein‐protein interactions (PPI) from the UniProt database^[^ ^13^ ^]^ to enrich protein functional representations and enhance network connectivity. To provide a more detailed characterization of protein biological functions, BioHG integrates Gene Ontology (GO) data from the UniProt database and pathway data from the Reactome database,^[^ ^14^ ^]^ enriching foundational and higher‐order biological information, respectively.

After determining the data sources, two aspects of edge design are defined: 1) drugs and cell lines are not directly connected. This design forces KGDRP to learn the drug response data through proteins, thereby allowing more comprehensive use of network information to enrich representations. 2) Regarding the relationship between proteins and cell lines, drawing inspiration from the work of Torras et al.^[^ ^15^ ^]^ proteins exhibiting expression values higher than the mean value of each cell line are used to establish the edges of the protein‐cell line relationship. By establishing this type of connection, transcriptomic data and biological knowledge are interconnected in a graph‐based structure. The links between these cell line nodes and protein nodes are assigned transcriptional expression values as edge weights. It should be noted that these edge weights are used for training sample generation but not used in message passing of the HGNN module. Overall, our BioHG contains 171103 nodes and 2354380 edges. Detailed information on its construction is provided in the experimental section.

#### 2.1.2 Overview of the KGDRP Approach

To facilitate phenotype screening across a broader range of chemical molecules, KGDRP introduces a linear function to convert the structural information of chemical molecules into biological representations. To effectively incorporate prior biological knowledge, KGDRP extends beyond relying solely on drug‐cell response data. It incorporates three additional predictors: the RNA expression predictor, the DTI predictor, and the biological process predictor. These auxiliary predictors enable KGDRP to capture inherent correlations and dependencies across diverse biological networks by learning multiple tasks simultaneously. For drug response prediction, KGDRP transforms the task from a reactive numerical prediction (regression) into a numerical comparison (classification) task. This task transformation is necessary because it allows the model to focus on distinguishing relative differences between drugs rather than predicting absolute response values. This approach reduces the model's dependency on specific experimental data, which can vary widely in quality and availability, and enhances its robustness. It also improves predictive performance by leveraging comparative data, which is often more reliable and less prone to overfitting. This strategy is also applied to the relationship between proteins and tumor cell lines, ensuring that the model can generalize well across different biological contexts. In summary, in KGDRP, BioHG serves as the heterogeneous graph structure that incorporates biomedical data, while the auxiliary tasks, such as RNA expression predictor, DTI predictor, and biological process predictor, are designed to enhance the model's ability to learn meaningful representations from BioHG.

#### 2.1.3 Performance on the Drug Response Prediction

To evaluate the performance of KGDRP, we conducted a comparison between KGDRP and six advanced drug response prediction methods on the Genomics of Drug Sensitivity in Cancer (GDSC) dataset. Here we categorized the models for drug response prediction into three types based on their feature sources. The first category includes deep learning models that primarily leverage gene expression profile and drug chemical structures as input features, such as DeepTTA^[^ ^16^ ^]^ and MLP. The second category comprises knowledge‐guided models, such as DEERS,^[^ ^17^ ^]^ Precily,^[^ ^18^ ^]^ and PathDNN,^[^ ^19^ ^]^ which utilize knowledge‐driven encodings to integrate biomedical information, enhancing interpretability and generalizability. The third category consists of network‐based models, represented by NetGP,^[^ ^8^ ^]^ which leverages biological knowledge such as PPI and DTI through network propagation. Detailed specifications of these baseline methods are provided in the Experimental Section. This comparison covered four realistic scenarios: warm, cold cell, cold drug, and cold both (as detailed in the Experimental Section, Figure S1, Supporting Information). The GDSC dataset, though extensive, lacks a comprehensive representation of the entire pharmacological landscape and does not cover all possible cancer cell lineages.^[^ ^20^ ^]^ Given these limitations, our focus is on evaluating the model's transferability in scenarios including cold cell, cold drug, and cold both. Three metrics have been used in the drug response prediction: Root Mean Square Error (RMSE), Pearson's Correlation Coefficient (PCC) and SCC, as detailed in the Experimental Section. The primary aim of our work is to enhance the generalization ability of drug response prediction. In realistic scenarios, such as wet‐lab experiments, the goal is often not to predict exact numerical values but to assess the relative ranking of responses, such as comparing with positive controls and negative controls. Therefore, SCC is used as the predominant metric, as it evaluates rank‐order correlation and is robust to non‐linear relationships and variations in scale.^[^ ^21^ ^]^

As indicated by the results shown in **Table** **1**, KGDRP exhibits a comparable predictive performance compared to six highly competitive methods. In the “cold both” scenario, which is crucial for precision medicine, KGDRP demonstrated significant improvements over the second‐best performing method (*RMSE_improvement_* =  0.169,  *PCC_improvement_* =  0.110,  *SCC_improvement_* =  0.120). This enhancement highlights KGDRP's effectiveness in leveraging biomedical information to improve phenotypic screening. We also investigated the impact of different biomedical graph configurations on drug response prediction performance (Table S2, Supporting Information), which demonstrates improved performance over the BioKG. This comparison result indicates that the predictive efficacy of a biomedical graph is not solely determined by the breadth of the data it includes but is significantly influenced by the relevance of the connections forged within it. The BioHG, with its curated approach, suggests that a more focused integration of data, which prioritizes the quality of interactions over their quantity, can yield a more biologically insightful and computationally efficient model. Regarding the “warm” scenario, it is observed that the DeepTTA method, utilizing solely gene expression and drug sequence data, demonstrates superior performance. This finding indicates that a deep learning model without auxiliary information is capable for drug response prediction when the test data lies within the domain of the training data. To explore the representation capacity of embeddings for cell lines, we employed the disease (tissue) information of cell lines as labels to calculate the clustering accuracy. As illustrated in Figure S2 (Supporting Information), the distribution of clustering accuracies for the 804 cell lines indicated that the embeddings derived from BioHG captured the cellular context more effectively. To explore the impact of the auxiliary predictors, we further conducted ablation experiments by removing each of the auxiliary tasks individually. The results shown in **Table** **2** indicated that KGDRP consistently outperformed its variants without these auxiliary predictors, demonstrating their contribution to the model's performance and highlighting the critical role of prior knowledge in improving prediction accuracy.

**Table 1:** Performance comparison on GDSC datasets under four scenarios.

Metrics	Scenarios	DeepTTA	MLP	DEERS	Precily	PathDNN	NetGP	KGDRP
RMSE	Warm	0.999 ± 0.009	1.073 ± 0.012	1.212 ± 0.020	1.290 ± 0.016	1.405 ± 0.011	**0.998 ±** **0.008**	1.019± 0.008
	Cold Cell	1.337 ± 0.030	1.353 ± 0.027	1.368 ± 0.034	1.775 ± 0.062	1.748 ± 0.069	1.326 ± 0.030	**1.323± 0.032**
	Cold Drug	2.510 ± 0.358	2.587 ± 0.292	2.623 ± 0.375	2.715 ± 0.240	2.948 ± 0.384	2.409 ± 0.285	**2.288 ±** 0.234^*^
	Cold Both	2.458 ± 0.312	2.497 ± 0.291	2.458 ± 0.312	2.753 ± 0.285	2.668 ± 0.417	2.343 ± 0.254	**2.174 ±** 0.223^*^
PCC	Warm	**0.928 ±** **0.001**	0.917 ± 0.002	0.892 ± 0.004	0.876 ± 0.003	0.869 ± 0.002	**0.928 ±** **0.001**	0.925 ± 0.001
	Cold Cell	0.869 ± 0.007	0.862 ± 0.006	0.859 ± 0.007	0.765 ± 0.015	0.793 ± 0.016	**0.869 ±** **0.006**	0.868 ± 0.006
	Cold Drug	0.424 ± 0.155	0.380 ± 0.129	0.294 ± 0.132	0.467 ± 0.125	0.177 ± 0.230	0.468 ± 0.083	**0.556 ±** 0.115^*^
	Cold Both	0.284 ± 0.141	0.346 ± 0.145	0.284 ± 0.141	0.433 ± 0.120	0.232 ±0.162	0.412 ± 0.130	**0.543 ±** 0.075^*^
SCC	Warm	**0.905 ±** **0.002**	0.891 ± 0.003	0.857 ± 0.004	0.836 ± 0.005	0.823 ± 0.002	0.904 ± 0.002	0.901 ± 0.002
	Cold Cell	0.822 ± 0.009	0.816 ± 0.008	0.812 ± 0.009	0.692 ± 0.020	0.723 ± 0.020	0.824 ± 0.008	**0.825 ±** **0.008**
	Cold Drug	0.377 ± 0.117	0.343 ± 0.120	0.274 ± 0.108	0.419 ± 0.134	0.182 ± 0.224	0.440 ± 0.075	**0.516 ±** 0.113^*^
	Cold Both	0.240 ± 0.121	0.282 ± 0.136	0.240 ± 0.121	0.376 ± 0.113	0.224 ± 0.143	0.357 ± 0.103	**0.496 ±** 0.085^*^

**Table 2:** Performance comparison on GDSC datasets under four scenarios.

Metrics	Scenarios	KGDRP_noRNA	KGDRP_noDTI	KGDRP_noBP
RMSE	Warm	1.109± 0.018*	1.107± 0.021*	1.112± 0.027*
	Cold Cell	1.433± 0.044*	1.400± 0.029*	1.425± 0.038*
	Cold Drug	2.401 ± 0.259*	2.446 ± 0.270*	2.434 ± 0.257*
	Cold Both	2.357 ± 0.231*	2.434 ± 0.264*	2.408 ± 0.181*
PCC	Warm	0.911 ± 0.003*	0.911 ± 0.004*	0.910 ± 0.005*
	Cold Cell	0.847 ± 0.009*	0.853 ± 0.006*	0.848 ± 0.008*
	Cold Drug	0.470 ± 0.133*	0.454 ± 0.147*	0.480 ± 0.138*
	Cold Both	0.452 ± 0.110*	0.430 ± 0.106*	0.427 ± 0.117*
SCC	Warm	0.881 ± 0.004*	0.881 ± 0.005*	0.880 ± 0.007*
	Cold Cell	0.795 ± 0.011*	0.802 ± 0.008*	0.796 ± 0.011*
	Cold Drug	0.430 ± 0.105*	0.411 ± 0.128*	0.433 ± 0.128*
	Cold Both	0.400 ± 0.086*	0.378 ± 0.089*	0.379 ± 0.103*

Values in bold indicate the best performance, while values underlined indicate the second‐best performance. * ± * denotes the mean and standard errors. An asterisk (*) denotes statistical significance compared to the second‐best performing method with a *p*‐value < 0.05, determined by the Wilcoxon signed‐rank test.

Values in bold indicate the best performance, while values underlined indicate the second‐best performance. * ± * denotes the mean and standard errors. An asterisk (*) denotes statistical significance compared to KGDRP with a *p*‐value < 0.05, determined by the Wilcoxon signed‐rank test. KGDRP_noRNA, KGDRP_noDTI and KGDRP_noBP indicate the KGDRP variant without the RNA expression predictor, DTI predictor, and the biological process predictor, respectively.

#### 2.1.4 Performance on the Preclinical Drug Response Prediction

Then, we conducted an exploration of the potential for transferring drug response models trained on cell lines to preclinical disease models, which is to evaluate the applicability of these models to a wider range of tumor heterogeneity scenarios. Patient‐derived tumor xenografts (PDTX) have emerged as invaluable tools in cancer research, bridging the gap between traditional pre‐clinical models and clinical applications. Specifically, PDTX models involve the implantation of patient‐derived tumor tissues into immunodeficient mice, allowing the propagation of the tumor in a microenvironment that retains essential features of the human disease. In this study, we assessed the transferability of these models using breast cancer PDTXs obtained from Project Biobank.^[^ ^22^ ^]^ The external test dataset comprised 1595 drug response data points, encompassing 20 PDTX breast cancer models and 101 distinct drugs. Two baseline methods were employed for comparison. The first is DeepTTA, which represents the drug response data that barely rely on drug and cell line information, demonstrating a robust and relatively strong predictive performance on the GDSC dataset. The second method is a modified version of KGDRP that excludes BioHG data. In this version, the auxiliary predictors are also removed since the embedding of external biological entities could not be achieved from the drug‐cell line graph.

**Figure 2:**
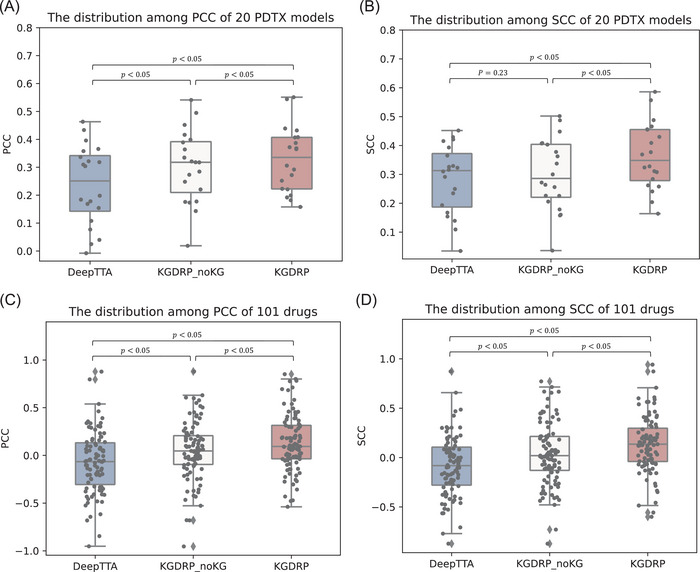
Transfer performance assessment from high‐throughput screens to pre‐clinical drug screening. A) and B) show the distribution of PCC and SCC for 20 PDTX models, respectively. These correlation coefficients are calculated by comparing model predictions to actual responses for each PDTX model. C) and D) represents the distribution among PCC and SCC of 101 drugs, respectively. These correlation coefficients are calculated by comparing the model predictions to the actual responses for each drug. Box plots show the median as the center lines, upper and lower quartiles as box limits, whiskers as maximum and minimum values, and dots represent outliers. Statistical significance was assessed using the Wilcoxon signed‐rank test.

To facilitate a realistic model performance comparison, we formulated two evaluation scenarios. First, we assessed the performance of model in successfully predicting drug sensitivities within each PDTX model. In this scenario, KGDRP outperforms other models, achieving an average PCC of 32.9% and an average SCC of 36.4% (**Figure** **2**A,B; Table S3, Supporting Information). Compared to DeepTTA, KGDRP demonstrated notable improvements of more than 10% in 11 out of 20 PDTX models. Similarly, the inclusion of BioHG data enhanced KGDRP's performance in 19 out of 20 PDTX models. This compelling evidence emphasizes the substantial impact of integrating prior biological knowledge on the generalization capacity of GNN models for drug response prediction. Then, we assessed the performance of model in effectively predicting PDTX model response to each drug, providing an in‐depth exploration of the potential for transferring knowledge in drug repurposing within the context of diverse molecular stratifications in cancer treatment. In this scenario, the complexity and heterogeneity of tumors posed challenges, leading to suboptimal performance for all models, as depicted in Figure 2C,D. KGDRP demonstrated superior robustness and predictive capability, achieving higher average PCC and SCC values compared to both DeepTTA (*PCC_improvement_* =  13.2%,  *SCC_improvement_* =  13.6%) and its variant without BioHG (*PCC_improvement_* =  8.3%,  *SCC_improvement_* =  9.8%). These results indicate that auxiliary biomedical data significantly enhance the accuracy and transferability of the KGDRP model in drug response prediction.

### 2.2 Phenotypic Data Empowers Prioritization of Therapeutics Targets Discovery

**Figure 3:**
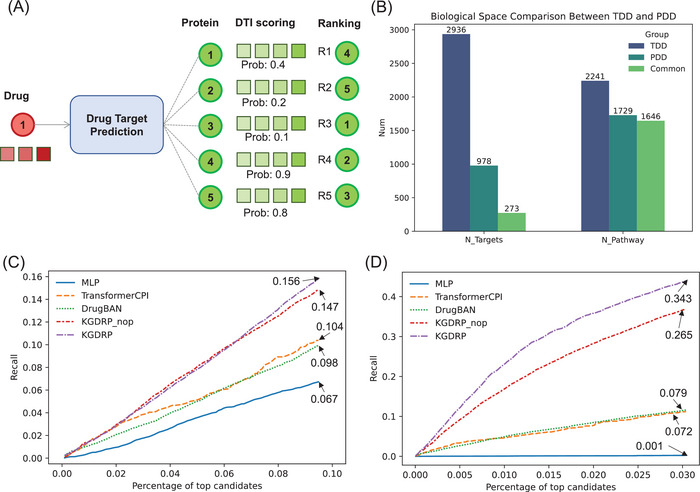
Phenotypic data empowers prioritization of therapeutics targets discovery. A) depicts a graphical representation of the target prioritization evaluation pipeline. B) The biological space comparison involves targets from TDD and PDD sources. The TDD and PDD groups indicate the number of targets and pathways derived from DrugBank and GDSC data, respectively. “Common” refers to the targets and pathways shared by both TDD and PDD sources. C,D) Comparison of ranking performance in the task of target prioritization for 1054 proteins and 16430 proteins, respectively.

Another primary goal in this study is to explore the potential effects of biomedical data integration for prioritization of therapeutics targets (**Figure** **3**A). Preceding investigations into the MoA of approved compounds, derived from phenotypic screenings, have compellingly exhibited the significance of phenotypic strategies as an avenue to broaden the horizons of the “druggable target space”.^[^ ^4^ ^]^ Here, we concentrate on utilizing phenotypic data within the KGDRP framework to enhance the recall of drug target predictions, thereby accelerating the identification of therapeutic targets. As depicted in Figure 3B, despite differences in the specific targets between TDD and PDD sources, the target‐related pathways are fundamentally the same. This finding indicates a strong potential for alignment in the biological space, which could facilitate the transfer of knowledge across datasets and enhance the generalizability of predictions in the TDD context.

#### 2.2.1 Performance on the Drug Target Prediction

To comprehensively evaluate the performance of target prioritization, we constructed a dataset of protein targets for each drug in the GDSC dataset from PubChem. Our target evaluation dataset comprises 9141 positive DTI pairs that are exclusive from the existing DTIs in the BioHG. Noted, the training dataset and validation set are random split from the existing DTIs in the BioHG and the sampled negative DTIs (see Training samples generation in Methods). Negative DTI pairs in the evaluation dataset were considered in two scenarios: first, by restricting targets to proteins with known DTIs, and second, by considering all proteins in curated biomedical networks as candidates. In the target prioritization evaluation pipeline, we first computed scores for DTI pairs involving all drugs and human proteins within the BioHG. For model comparison, we focused on two key factors for selecting baseline methods: the types of training data used and the representation algorithms employed. We considered two types of methods: 1) descriptor‐based methods and 2) sequence‐based methods. MLP is categorized as a descriptor‐based method, as it relies on pre‐calculated representations for both drugs (Morgan Fingerprint) and proteins (ProteinBERT embedding). In contrast, TransformerCPI and DrugBAN are sequence‐based methods, as they learn drug and protein representations directly through end‐to‐end model training for DTI prediction. BioHG‐based methods were excluded from the baseline comparison since some drugs identified through PDD approaches lack connections within the BioHG. Consequently, we employed three effective DTI prediction methods as baseline models: MLP, TransformerCPI,^[^ ^23^ ^]^ and DrugBAN.^[^ ^24^ ^]^ Additionally, to assess the impact of phenotypic data on KGDRP, we also evaluated a variant of KGDRP that excludes drug response data, referred to as KGDRP_nop.

When targeting proteins within known DTIs, the predicted target protein typically matches those identified by the established DTI prediction model, suggesting a scenario related to drug repurposing. Conversely, when considering all human proteins in the biomedical network, the predicted target protein could be novel to the drug target prediction model, indicating a scenario more aligned with novel therapeutic target discovery. In the scenario of drug repurposing (Figure 3C), KGDRP achieved the best recall of 0.156 among the top 10% candidates. Similarly, in the scenario of novel therapeutic targets discovery (Figure 3D), KGDRP achieved the best recall of 0.343 in the top 3% candidates, showing an 8% improvement over KGDRP_nop. These results demonstrate that incorporating phenotypic data into the KGDRP framework enhances target discovery efficacy. Additionally, compared with descriptor‐based methods like MLP and sequence‐based approaches such as TransformerCPI and DrugBAN, knowledge‐guided approaches showed significant improvements in both scenarios. For instance, in the scenario of drug repurposing, KGDRP outperformed the best performing sequence‐based method TransformerCPI by 5%. Similarly, in the scenario of target discovery, KGDRP outperformed the best performing sequence‐based method DrugBAN by 26%. This finding consistently aligns with our previous research, reinforcing the inherent advantages of knowledge‐guided DTI prediction methods in the context of drug target discovery.^[^ ^25^ ^]^ It is observed that the MLP model demonstrated poor performance compared to other methods. The result is primarily influenced by the representations used for both chemicals and proteins, with protein representation playing a particularly critical role in the target discovery experimental setting. Specifically, the protein representations for MLP were derived from ProteinBERT as 1024‐dimensional embeddings. The poor performance of MLP can primarily be attributed to the ineffectiveness of this embedding in capturing the biological and binding properties necessary for DTI prediction.

#### 2.2.2 KGDRP Facilitated Drug Mechanism Analysis

Subsequently, we demonstrate the drug mechanism analysis pipeline based on KGDRP through two drug target prediction cases. The selection of these two cases was based on the number of drug‐target interactions contained in the heterogeneous biomedical network. The first case centers around the drug lestaurtinib, wherein a portion of the target proteins corresponding to the representative drug has been integrated into the heterogeneous biomedical network. Lestaurtinib, denoted as DB06469 in the DrugBank database,^[^ ^26^ ^]^ an orally administered and highly selective inhibitor of receptor protein tyrosine kinases.^[^ ^27^ ^]^ Lestaurtinib is a representative case wherein a portion of its associated drug targets is integrated into our BioHG. In our curated DTI dataset, lestaurtinib have 348 verified target proteins and only 2 DTIs exist in our BioHG. KGDRP exhibits remarkable performance for this drug, achieving an 94% accuracy (47 hits in the top 50). In contrast, other methodologies such as MLP, TransformerCPI, DrugBAN, and KGDRP (without drug response data) identified no more than 50% of the hits (Table S4, Supporting Information).

**Figure 4:**
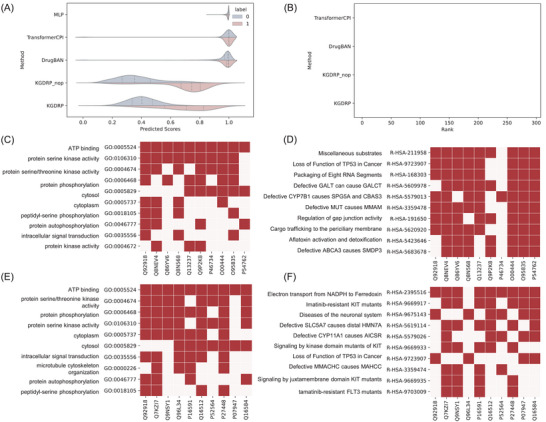
KGDRP facilitated drug mechanism analysis. A) represents the prediction distribution of all human targets specifically for the drug lestaurtinib. B) represents the ranking distribution of the top 300 predictions for the compound PF562271. C) and D) represent heatmaps visually convey the results of the GO analysis and pathway analysis for lestaurtinib. E) and F) represent heatmaps visually convey the results of the GO analysis and pathway analysis for PF562271.

To delve deeper into the detailed ranking results, we conducted a visual examination of the predictive score distribution generated by these five methods. As depicted in **Figure** **4**A, a clear distinction emerges in the distribution of positive and negative outcomes when employing BioHG‐based methods. In contrast, for sequence‐based methods, the negatives are scored as high as positives. A potential explanation for this phenomenon lies in the limitations of sequence‐based DTI prediction methods in capturing the full biological context. Specifically, the complexity biological system sometimes leads to a situation where the sequence information alone is not sufficient to accurately distinguish between interacting and non‐interacting DTI pairs.^[^ ^28^ ^]^ For instance, factors like post‐translational modifications and tissue‐specificity may not be captured by sequence‐based methods. In the scope of our experiments encompassing over 16000 human protein candidates, the majority of protein space remains unexplored due to high costs, resulting in suboptimal performance of sequence‐based models. Moreover, when comparing the ranking results among the five methods, it becomes evident that the top 10 scored proteins, which have been successfully validated by KGDRP, do not overlap with the results generated by the other methods (Table S5, Supporting Information). This discovery indicates the unique advantages of phenotypic data in the prioritization of therapeutic target discovery. To explore the mechanism of lestaurtinib, we present the top 10 gene ontology (GO) terms that correlated with the above top 10 ranked protein targets of the drug through the BioHG (Figure 4C). The results unveil a notable association between ATP binding and the targets of lestaurtinib. This finding aligns seamlessly with the known Mode of Action (MoA) of lestaurtinib, as it functions as an ATP‐competitive dual inhibitor targeting JAK2 and FLT3 kinases.^[^ ^29^ ^]^ Subsequently, we computed pathway‐protein association scores, relying on the biological process predictor within KGDRP (Figure 4D). This approach was necessitated by the relative scarcity of prior pathway information within the BioHG. At the forefront of our rankings, we find the pathway titled “Miscellaneous substrates”, a category encompassing a quarter of the 57 human Cytochrome P450 (CYP) enzymes capable of catalyzing CYP‐like reactions in vitro. This discovery harmonizes with existing evidence indicating lestaurtinib inhibitory effects on CYP2C8 and CYP3A activity.^[^ ^30^ ^]^ Furthermore, the association between lestaurtinib and the “Loss of Function of TP53 in Cancer” pathway is substantiated by a recent study that lestaurtinib could induces cell cycle arrest, apoptosis, and activates the TP53 superfamily within medulloblastoma cells.^[^ ^31^ ^]^

The second case is PF562271, a potent ATP‐competitive and reversible FAK and Pyk2 kinase inhibitor.^[^ ^32^ ^]^ PF562271 represents the drug that none of the information on the drug is involved in the biomedical knowledge except drug response information. In our curated DTI dataset, lestaurtinib have 54 verified target proteins and no related DTIs exist in our BioHG. In this case, KGDRP demonstrates superior performance with six hits within the top 50 ranked proteins (Table S4, Supporting Information; Figure 4B). In contrast, the other four baselines identified no hits or only a few (Table S6, Supporting Information). These results emphasize the incredible benefits of incorporating phenotypic data and leveraging BioHG in the discovery of therapeutics targets. The analysis of GO terms associated with PF562271 (Figure 4E) aligns well with a prior study that demonstrated a robust correlation between heightened FAK expression and the phosphorylation status observed in aggressive human tumors.^[^ ^32^ ^]^ Moreover, delving into the pathway analysis of PF562271 (Figure 4F) offers intriguing insights into a potentially novel MoA. Notably, the pathway analysis suggests a potential impact of PF562271 on Imatinib‐resistant KIT mutants, a finding that harmonizes with an existing study reporting that FAK inhibitor treatment in imatinib‐resistant cells bearing KIT mutations enhances imatinib‐induced apoptosis.^[^ ^33^ ^]^ Our analysis results indicate the considerable potential for knowledge guided GNN methods for therapeutics target discovery and mechanism analysis.

### 2.3 Zero‐Shot Evaluation by Drug Repurposing for COVID‐19

Introducing biomedical data through graph‐based methods effectively address several limitations of phenotype‐based drug screening in zero‐shot scenarios, such as limited availability of known drug response data, restricted chemical space, and the exploration of drug mechanisms. In this section, we aim to demonstrate the advantages and potential of a computational framework that leverage biomedical knowledge to address challenges such as limited training data, constrained chemical space and the need for mechanism exploration. By integrating expression profiling of SARS‐CoV‐2 infected cell models,^[^ ^34^ ^]^ KGDRP could facilitates the generation of a meaningful representation of lung samples for subsequent phenotypic screening.

#### 2.3.1 Performance on the Phenotypic Drug Repurposing

**Figure 5:**
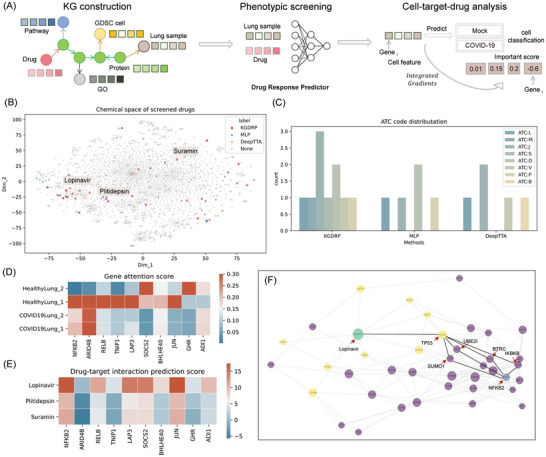
Zero‐shot evaluation by drug repurposing for COVID‐19. A) Illustrates the repurposing pipeline, encompassing knowledge graph construction, phenotypic screening, and cell‐target‐drug analysis. B) visualizes the chemical space occupied by the screened drugs. C) represents the distribution of ATC codes for successfully screened drugs using KGDRP, MLP, and DeepTTA, respectively. D) presents attention scores for the top 10 genes associated with COVID‐19 across four lung samples. E) depicts the predictions of drug‐target interactions involving the top 10 genes related to COVID‐19 for three representative repurposed drugs. F) Network describing the relationship between Lopinavir and NFKB2. In this visualization, the green node symbolizes the drug Lopinavir, while the blue node represents the gene NFKB2. Yellow nodes indicate the predicted targets of Lopinavir, and the remaining purple nodes represent proteins within the network. Notably, the node size is determined by betweenness centrality, where larger betweenness centrality corresponds to a larger node size.

Leveraging the GDSC dataset for training drug response predictors, we repurposed 7070 drugs against four lung samples, comprising two healthy and two SARS‐CoV‐2 infected samples (**Figure** **5**A). The representation of these four lung samples, based on KGDRP and expression profiles incorporated into BioHG, was used to obtain drug response scores with 7070 drugs using an MLP model trained on the GDSC dataset. Subsequently, the screening strategy involved identifying drugs with high scores for SARS‐CoV‐2 infected samples and low scores for healthy samples (see methods). To exhibit the advantages of KGDRP, we conducted additional drug repurposing using two baselines: MLP and DeepTTA.

For KGDRP, we obtained 29 drug candidates, and we identified 10 drugs (34.5%) with supporting evidence from the literature and databases like PubChem (Table S7, Supporting Information). In contrast, the successfully validated ratio for MLP and DeepTTA was 18.8% (6/32) and 13.8% (4/29), respectively. Compared with the two baselines, KGDRP demonstrated a significantly higher success rate in identifying potential drug candidates, illustrating its effectiveness in harnessing BioHG for phenotypic drug screening. One potential explanation for the prowess of BioHG‐based computational methods lies in the advantages derived from a more extensive chemical space provided by the wealth of information within the BioHG. Delving into the chemical space distribution among various methods, we visually represented the chemical space of 7070 repurposed drugs through t‐SNE dimension reduction of molecular fingerprints, as illustrated in Figure 5B. Notably, the chemical space distribution exhibited variations among the three methods. Lopinavir, originally used for HIV treatment, emerged as the sole drug successfully predicted by all three methods, exhibiting inhibitory effects on both SARS‐CoV and SARS‐CoV‐2 replication in vitro.^[^ ^35^ ^]^ Plitidepsin, predicted by both KGDRP and MLP, is a compound with antitumor, antiviral, and immunosuppressive properties, demonstrating significant potential in inhibiting SARS‐CoV‐2 replication, surpassing remdesivir by 27.5 times in similar cell types.^[^ ^36^ ^]^ Another repurposed drugs, Suramin, only identified by KGDRP, exhibited distinct chemical spaces from the other drugs, while also showing promising anti‐SARS‐CoV‐2 effects.^[^ ^37^ ^]^ Further analysis involved comparing the diversity of repurposed drugs by calculating the distribution of Anatomical Therapeutic Chemical (ATC) codes. Figure 5C reveals that KGDRP encompasses seven different ATC types, in contrast to the other two methods, which only have four types. This result suggests the remarkable ability of KGDRP to discover a broader range of drug types effective against COVID‐19.

#### 2.3.2 KGDRP Facilitated Cell Line‐Target‐Drug Analysis

More significantly, by harnessing the knowledge graph and computational modules within KGDRP, we empower the execution of cell line‐target‐drug analysis, thereby facilitating the discovery of drug mechanisms. In the model training phase, a cell line classification predictor embedded within KGDRP, utilizing MLP, categorized the 78 cell lines as either infected or not infected. This process enabled the application of integrated gradients,^[^ ^38^ ^]^ shedding light on the correlation between a model's predictions and its features. Given that the input feature for the cell line classification model comprises gene expression profiles, the contribution score assigned to each feature serves as a vital metric, representing the significance of each gene against COVID‐19. In alignment with this, Figure 5D represents the distribution of attention score of top 10 genes against the four lung samples. Notably, these top‐ranked genes demonstrate established associations with COVID‐19, supported by both literature evidence and an Open Targets search (Table S8, Supporting Information). Noteworthy among these genes are NFKB2, RELB, and TNIP1, actively participating in the NF‐κB (Nuclear Factor Kappa B) pathway. The observed activation of NF‐κB in response to SARS‐CoV‐2 results in an escalated inflammatory response, a crucial factor in the virus's pathogenesis.^[^ ^39^ ^]^ This activation highlights the NF‐κB pathway as a promising target for COVID‐19 treatment.^[^ ^40^ ^]^ By employing the integrated cell line and drug target predictors within KGDRP, we could elucidate the drug mechanism within the BioHG. This involves merging the results of drug‐target interaction (DTI) predictions and cell line ‐gene association predictions through network analysis. To gain a more profound understanding of how Lopinavir influences the NFKB2 gene, we utilize the DTI predictor to identify predicted targets.

Consequently, we construct a subgraph that encompasses Lopinavir, its predicted targets, NFKB2, and the proteins situated along the shortest path among these nodes. Utilizing the network analysis metric, betweenness centrality, we characterize the importance of each gene in elucidating the relationship between Lopinavir and NFKB2.^[^ ^41^ ^]^ The DTI prediction results, as depicted in Figure 5E, suggest a potential impact of Lopinavir on NFKB2. To further explore the specific mechanism between the screened drug and the cell line ‐related gene, we constructed a subgraph illustrating the relationship between Lopinavir and NFKB2 shown in Figure 5F. The primary objective of this step is to identify essential proteins that mediate the connection between drug‐target interactions and disease‐gene associations. In pursuit of this, we computed the shortest paths connecting the predicted targets of Lopinavir and NFKB2. Subsequently, we assessed the betweenness centrality for each node within the subgraph. TP53, predicted as a target of Lopinavir and exhibiting the highest betweenness centrality, has been reported to undergo reciprocal balance alterations with NF‐kB upon SARS‐CoV‐2 infection.^[^ ^42^ ^]^ Consequently, we infer that the mechanism of Lopinavir against SARS‐CoV‐2 infection may involve targeting TP53, leading to its activation and influencing the reciprocal balance with NF‐kB. These interactions collectively contribute to a potential antiviral and immunomodulatory response, emphasizing the multi‐faceted impact of Lopinavir in combating COVID‐19.

## 3 Discussion

In this study, we develop a generalized framework KGDRP leveraging multimodal biomedical data for therapeutic molecule discovery. KGDRP utilizes graph‐based data fusion techniques to integrate transcriptomic data, chemical structure information, and biological network data, enabling a comprehensive characterization of biological entities. While the modeling of heterogeneous graphs for drug discovery tasks remains challenging due to the unbalanced distribution of different types of edges (or relations) and the presence of considerable noises in biological data, the robust and generalized performance of the KGDRP is achieved through a well‐designed structured BioHG coupled with a multi‐constraints training strategy. This approach effectively enhances the performance and interpretability of a range of downstream tasks, which is the model's capacity to provide biologically meaningful explanations for its predictions.

The performance of KGDRP was evaluated in four scenarios, including drug response prediction, preclinical drug screening, drug target prediction, and zero‐shot phenotypic drug screening. The experiment results indicate that through graph‐based deep learning fusion of multimodal biomedical data, KGDRP significantly improves drug response prediction between drugs and tumor cell lines in cold start scenarios. Furthermore, phenotype data could also greatly enhance the prediction performance of drug targets. We consider KGDRP as a sustainable computational framework, and a promising future direction is to continue incorporating different omics data, such as single‐cell omics and metabolomics, to expedite the exploration of disease mechanisms and target discovery. However, this necessitates further exploration on the fusion for different modalities of omics data. While our work demonstrates the potential of BioHG as a feasible approach, constructing heterogeneous graphs to effectively integrate diverse biomedical data remains an urgent issue to address. For instance, while we could model the relationship between cell lines and genes as edges between two types of nodes, further research is needed to explore whether edges require direction and how to introduce weights. Looking ahead, leveraging large foundation models from different modalities offers a promising avenue to enhance knowledge graph construction and utilization. For example, multimodal foundation models trained on diverse omics data, such as single‐cell transcriptomics, proteomics, and imaging data, could provide robust embeddings and insights that better inform graph structures and relationships. Integrating these models could enable more comprehensive and context‐aware knowledge graphs, ultimately improving their ability to facilitate downstream biomedical applications.^[^ ^43^ ^]^

We further evaluated the generalization, transferability, and interpretability of the KGDRP framework. When we applied the model trained on the laboratory‐based GDSC dataset to predict preclinical PDTX datasets, KGDRP exhibited promising performance in drug screening scenarios. However, we also observed that the performance may still not meet expectations, which could be a bottleneck issue in cancer treatment, where activity results obtained in laboratory research often fail to reproduce expected outcomes in clinical settings. To address this issue, we can further explore the following two aspects: 1) modeling the relationship between cell lines and genes can be approached from the perspective of addressing inevitable noise and bias in biological experiments. Training data always contain noise and bias due to unavoidable experimental variations. Rather than directly correcting these experimental biases through design methods, introducing omics data of different types, levels, and sources may be a more practical direction. Deep learning models, due to their powerful learning capabilities, often tend to learn incorrect assumptions from limited, noisy, and biased data. However, if heterogeneous data are based on the same biological hypotheses and can be reasonably used for model training, the model may learn correct assumptions to achieve collaborative predictive performance. Although the experiments in this work partially support this inference, strong support from biological experiments is lacking and requires further validation in subsequent work. 2) integrating transfer learning methods when applying model across datasets, where a portion of the data can be appropriately used as training samples for few‐shot learning. Some work has already been conducted on transfer learning based on neural network computational frameworks.^[^ ^44^ ^]^ However, besides considering the transfer of task data, computational methods should also fully leverage the heterogeneous biomedical data to enhance the generalization ability.

In emergency situations, particularly during pandemics like COVID‐19 that cause severe damage to human health, there is a pressing need to rapidly phenotype‐based drug screening. For rapidly evolving infectious diseases, two challenges are the unknown target proteins and the absence of known training data. Therefore, computational models need to rely on phenotype data, such as transcriptome data, for zero‐shot drug screening prediction. In this study, even in the absence of COVID‐19‐related drug response training data, KGDRP successfully identified reliable phenotype drugs. Benefiting from the construction of a biomedical heterogeneous network and multiple predictor modules in KGDRP, we were able to analyze genes, drug targets, and drug mechanisms related to COVID‐19. Overall, the KGDRP framework serve as a robust infrastructure for the seamlessly integration of PDD, TDD and BioHG, advancing therapeutic molecule discovery.

## 4 Experimental Section

### The KGDRP Framework

KGDRP harnesses heterogeneous Graph Neural Networks (GNNs) in a multi‐modal fashion. This end‐to‐end framework comprises three essential components: 1) the construction of a biomedical knowledge graph, 2) the generation of training samples tailored for knowledge‐guided constraints, and 3) heterogeneous message passing on BioHG.

### Biomedical Knowledge Graph Construction

To establish the relationship between cell lines and protein targets, we introduce Protein‐Cell line Associations from the Genomics of Drug Sensitivity in Cancer (GDSC) dataset.^[^ ^45^ ^]^ For alignment, the gene IDs used in expression are converted into Uniprot IDs via https://www.uniprot.org/id‐mapping. In cases where automated conversion is not feasible, manual conversion is employed. To connect protein targets and drugs, we include Drug‐Target Interactions from the DrugBank Database.^[^ ^26^ ^]^

For drug‐related information, the standardization of drug identifiers was carried out by SMILES mapping, with a preference for prioritizing DrugBank IDs. Simultaneously, chemical structures were manually from PubChem,^[^ ^46^ ^]^ thus enriching our drug‐related dataset. Another noteworthy aspect of our BioHG lies in its comprehensive incorporation of proteins, including enzymes, receptors, and other essential components. To enable robust representation and enhance model interpretability, the BioHG is enriched with pathway information. We gathered knowledge of proteins, identified by their Uniprot IDs, in the form of pathways, and PPIs. This extensive protein‐centric information framework facilitates the understanding of processes related to these biological entities. To explore the inherent mechanism of cellular context, our BioHG incorporates pathways sourced from the Reactome database. In total, our BioHG contains 171103 nodes and 2354380 edges.

### Training Samples Generation

Notably, to seamlessly integrate the RNA expression data into this framework, we conceptualized individual cell lines as distinct nodes. In this innovative approach, the connections linking a cell line to a gene effectively encode the pertinent expression information. Subsequently, we incorporated four distinct sets of relational data comprising RNA expression data, drug response data, DTI, and protein‐pathway associations to generate tailored training samples using the BioHG as a foundation. For RNA expression and drug response data, we strategically reformulated the task from value fitting to value comparison. This approach enhances the model's generalization capability, as these data types are inherently subject to fluctuations driven by experimental conditions. Regarding the two categories of interaction data (DTI and protein‐pathway associations), we frame them as binary classification tasks and employ network‐oriented negative sampling strategies across the BioHG, drawing inspiration from the work of Ayan Chatterjee et al.^[^ ^47^ ^]^ Specifically, by calculating the shortest paths between individual protein nodes and drug nodes across DTI network, we select the nodes of protein distanced significantly from the corresponding drug node as negative samples for DTI. A similar methodology is employed for generating negative samples of protein‐pathway associations.

### Heterogeneous Message Passing

Ultimately, a heterogeneous GNN‐based model is crafted to facilitate advanced representation learning leveraging DGL python package (https://www.dgl.ai). With an awareness of the challenge posed by the cold start problem in phenotypic screening, we employ the GraphSAGE layer for efficient message propagation.^[^ ^48^ ^]^ For edge type of protein‐protein interaction, protein‐pathway association, protein‐gene ontology association and drug‐target interaction, we utilized GraphSAGE for message passing. The convolutional results from different edge types are aggregated using summation. The neighborhood aggregation operation for the GraphSAGE layers can be expressed as follows:

(1) $$h_{N\left(V_{i}\right)}=\mathrm{Aggr}\mathrm{egat}\mathrm{or}\;\left(\left\{h_{v_{j}},\forall_{v_{j}}\varepsilon N\left(v_{i}\right)\right\}\right)$$

(2) $$h_{v_{i}}^{\prime }=\sigma \left(W\cdot \mathrm{CONCAT}\left(h_{v_{i}},h_{N\left(v_{i}\right)}\right)\right)$$

where $h_{N(v_{i})}$ is the embeddings of the neighboring nodes, *N*(*v_i_*) represents the neighbors of node *v*, *Aggregator* is the aggregation function, σ is the activation function, *W* is a learnable weight matrix and CONCAT denotes concatenation. Specifically, for the edge type of protein‐cell association, we employed GCN for message passing. The message aggregation for the GCN layers can be expressed as follows:

(3) $$h_{v_{i}}^{\prime }=\sigma \left(W\cdot \sum_{v_{j}\;\varepsilon \;N\left(v_{i}\right)}\frac{1}{\sqrt{\deg\left(v_{i}\right)\cdot \deg\left(v_{j}\right)}}\cdot W\cdot h_{v_{j}}\right)$$

where deg(i) is the degree of node *v_i_*. The model incorporates node embeddings for various entity types, including proteins, drugs, pathways and cell lines. These embeddings are trainable and capture the underlying features of each entity type. Specifically, the embeddings for drug and cell line nodes are initialized with a 1024‐bit Morgan fingerprint using RDKit (https://www.rdkit.org) and gene expression features, respectively. One challenge in merging PDD and TDD is that the drugs involved in these two approaches may not overlap, resulting in some drugs lacking connections within the biomedical heterogeneous graph. To address this issue, KGDRP introduces a transformation function for drug nodes, which learns a mapping function from molecular fingerprints to drug representations.

(4) $$h_{drug}=W_{drug}\cdot x_{drug}$$

where *h_drug_* is the drug features, *W_drug_* is a learnable weight matrix for the learner transformation and *x_drug_* is the molecular fingerprint of drugs. In contrast, the embeddings for protein, pathway, and gene ontology nodes are initialized randomly. Given that direct message passing doesn't occur on drug and cell line nodes, we introduce three auxiliary predictors in the form of Multi‐Layer Perceptron (MLP) decoders, including the RNA expression predictor, drug target predictor, and biological process predictor. These predictors collectively guide the model to precise updates, effectively enhancing its capacity for superior generalization and heightened robustness. After attaining embeddings for biomedical entities, we employ MLP regressor to train the drug response prediction model. This approach enables us to harness the information more effectively within drug response data, capitalizing on the enhanced representations crafted earlier.

### Training Setup for KGDRP

KGDRP employs a Heterogeneous Graph Neural Network (HGNN) as its foundational model architecture. Specifically, each layer of the GNN includes parameters such as the dimensionality of input features, the dimensionality of hidden layer features, node representation aggregation method, and feature dropout rate.

During the model training phase of KGDRP, we update model parameters using training sets segmented based on different scenarios from the GDSC dataset. Model selection and adjustment are performed using a validation set, and the final evaluation of model generalization ability and performance is conducted on a test set. In this study, we adopt a 20‐fold cross‐validation approach for model evaluation. To optimize model performance, we utilize the Optuna package for hyperparameter selection.^[^ ^49^ ^]^ We employ the Adam optimizer as the model's optimization algorithm, setting the learning rate to 0.001 and the weight decay rate to 10^−6^ to minimize the loss function.

The model's loss function consists of four parts: binary cross‐entropy loss for the expression levels between tumor cell lines and two different genes, denoted as *loss*_pc_; binary cross‐entropy loss for the responsiveness levels between tumor cell lines and two different drugs, denoted as *loss*_dc_; binary cross‐entropy loss for the interactions between drugs and target proteins, denoted as *loss*_dpi_; and binary cross‐entropy loss for the interactions between proteins and biological pathways, denoted as $loss_{pro\_path}$. Binary cross‐entropy loss is calculated using the binary_cross_entropy_with_logits function from the Pytorch package, as shown in the following:

(5) $$loss_{BCE}=-\frac{1}{N}\sum_{i=1}^{N}\left(y_{i}\cdot log\sigma \left(x_{n}\right)+\left(1-y_{i}\right)\cdot log\left(1-\sigma x_{n}\right)\right)$$

where *N* is the number of samples, *y_i_* is the true label value, *x* is the model's predicted output, and σ is the sigmoid function that maps output values to the (0, 1) interval. The final loss function can be expressed as the following:

(6) $$\begin{array}{ccc} loss & = & w_{\mathrm{pc}}\times loss_{\mathrm{pc}}+w_{\mathrm{dc}}\times loss_{dc}+w_{\mathrm{dpi}}\times loss_{dpi}\\ & & +w_{pro\_path}\times loss_{pro\_path} \end{array}$$

where *w_pc_*, *w_dc_*, *w_dpi_* and $w_{pro\_path}$ are the weights corresponding to the respective loss, used to adjust the contribution of each loss in the total loss, obtained through hyperparameter tuning.

We employed a Bayesian optimization strategy to systematically explore a range of hyperparameters. The hyperparameters we tuned are listed in Table S9 (Supporting Information). A separate validation set was used to evaluate the performance of different hyperparameter combinations, with the primary evaluation metric being the SCC of the validation set. To prevent overfitting, we incorporated early stopping during training. If the validation SCC did not improve for a predefined number of epochs, training was stopped. The final model was then evaluated on the test set.

### Experimental Setup and Benchmarking for Drug Response Predictions

Six advanced drug response prediction methods are used for performance comparison.

1. DeepTTA: An end‐to‐end deep learning model that utilizes a Transformer to encode drug SMILES sequences and neural networks to extract tumor cell features. Initially, the SMILES sequence of drug molecules is parsed into distinct chemical substructures. For the tumor cell lines, features are extracted from gene expression data using a four‐layer neural network. Finally, the high‐level features of both the drug and transcriptomic data are combined and input into a drug response prediction model, which is a neural network consisting of four fully connected layers.
2. MLP: A common neural network that captures non‐linear relationships between drug descriptors and gene expression data.
3. DEERS is a deep neural network‐based recommendation system consisting of three main components: a drug autoencoder, a tumor cell line autoencoder, and a feedforward neural network. The drug features are represented by the biological expression profile of each drug molecule across 294 kinases, which corresponds to inhibition activity values, resulting in a feature vector of dimension 294. The tumor cell line features are represented by the corresponding gene expression matrix, with a dimension of 241. The drug autoencoder and the tumor cell line autoencoder share the same architecture, with each encoder and decoder having a 128‐dimensional hidden layer.
4. Precily: Utilizes SMILES‐based drug representations, pathway enrichment scores, and deep learning to improve predictions. The chemical structure of anticancer drugs is represented numerically using the SMILESVec method, which converts the SMILES strings of drugs into numerical representations. Gene expression data is processed through the MSigDB database platform, where biological pathway analysis is used to calculate the pathway enrichment scores for 1329 classical pathways based on the gene expression of tumor cell lines. The prediction model is a feedforward neural network with three hidden layers.
5. PathDNN: Introduces pathway‐specific layers informed by KEGG pathways, enhancing interpretability and prediction performance. PathDNN introduces a pathway layer into the neural network architecture, where biological pathways are represented as nodes, connected to the gene nodes in the input layer. Each neuron in the input layer corresponds to a gene. The pathway layer consists of 323 biological pathways from the KEGG pathway database, represented by 323 neurons. The connections between the input layer and the pathway layer are determined by the associations between genes and pathways, where a value of 1 indicates an association and 0 indicates no association. After the pathway layer, two fully connected hidden layers and an output layer follow.
6. NetGP applies network propagation to extract gene perturbation profiles informed by PPI and DTI. NetGP uses a network propagation method to extract gene perturbation scores containing drug mechanism information. These genes are ranked based on their perturbation scores, and the ranked genes are then input into a multilayer perceptron to generate a fixed‐dimensional vector, which serves as a supplement to the heterogeneous biomedical information. It's important to highlight that NetGP can be seamlessly integrated with various approaches, employing a dimension‐fixed vector generated through network propagation on a PPI network. For our investigation, we opt to combine NetGP with DeepTTA, as it demonstrates the most superior performance.

The drug response data is sourced from the GDSC database. GDSC is an international research project aimed at understanding cancer sensitivity and resistance to drugs, helping to reveal key biological mechanisms in cancer treatment by utilizing large‐scale cancer cell line drug sensitivity data and molecular feature data, thus providing a research basis for personalized therapy and drug development. Adhering to the dataset processing pipeline outlined by Minwoo Pak et al.,^[^ ^8^ ^]^ we employ a 20‐folds cross‐validation methodology for model evaluation. The drug response dataset utilized in this study comprises 227 drugs, 804 cell lines, and a total of 168244 drug response values. Each individual cell line is characterized by a 978‐dimensional feature set derived from the GDSC dataset (https://www.cancerrxgene.org). The 978 features correspond to the expression levels of genes selected based on the LINCS L1000 landmark genes.^[^ ^50^ ^]^ During the process of BioHG construction, we undertook data mapping by translating gene IDs into corresponding protein IDs using Uniprot ID references.^[^ ^51^ ^]^ In the context of a regression task for drug response prediction, three metrics are used for model predictive performance evaluation. First, the root mean square error (RMSE) serves as a measure to quantify accuracy and can be expressed as the following:

(7) $$RMSE=\sqrt{\frac{1}{n}\sum_{i}^{n}{\left(y_{i}-\hat{y_{i}}\right)}^{2}}$$

where $\hat{y_{j}}$ represents the predicted drug response value by the model, *y_i_* denotes the drug response value in the dataset, and *n* represents the number of samples in the test set. Second, the PCC is utilized to gauge the strength of the linear relationship between two continuous variables. PCC could be expressed as the following:

(8) $$PCC=\frac{\sum_{i}^{n}\left(y_{i}-\hat{y_{i}}\right)\left(x_{i}-\hat{x_{i}}\right)}{\sqrt{\sum_{i}^{n}{\left(y_{i}-\hat{y_{i}}\right)}^{2}}\sqrt{\sum_{i}^{n}{\left(x_{i}-\hat{x_{i}}\right)}^{2}}}$$

In this context, $\hat{y_{i}}$ and $\hat{x_{i}}$ represent the predicted drug response values for drugs *x* and *y* by the model, *y_i_* and *x_i_* denote the drug response values for drugs *i* and *j* in the dataset, and *n* represents the number of samples in the test set. The *PCC* ranges from ‐1 to 1, where 0 indicates no linear relationship, 1 indicates a perfect positive correlation, and ‐1 indicates a perfect negative correlation.

Lastly, the SCC is enlisted to assess the potency and direction of the monotonic relationship between two variables. SCC could be expressed as the following:

(9) $$SCC=\frac{\sum_{i}^{n}\left(r\left(y_{i}\right)-r\left({\hat{y}}_{i}\right)\right)\left(r\left(x_{i}\right)-r\left({\hat{x}}_{i}\right)\right)}{\sqrt{\sum_{i}^{n}{\left(r\left(y_{i}\right)-r\left({\hat{y}}_{i}\right)\right)}^{2}\sqrt{\sum_{i}^{n}{\left(r\left(x_{i}\right)-r\left({\hat{x}}_{i}\right)\right)}^{2}}}}$$

Unlike the PCC, which focuses on linear associations, the SCC evaluates the alignment of rank or order similarities between pairs of values in the two variables. During the model training phase, each training pairs of the four types generated in the second step of KGDRP undergoes a sampling process with varying data sizes, guided by hyperparameter optimization. This step aims to accelerate training while concurrently bolstering generalization capabilities. To determine the optimal parameter configuration, we employed Bayesian optimization, specifically utilizing the Optuna Python package (version 2.10.0).^[^ ^49^ ^]^ The specific hyperparameter configurations is shown in Table S10 (Supporting Information).

To ensure a comprehensive assessment, we conduct model comparisons across four scenarios: “warm”, “cold cell”, “cold drug” and “cold both”. A “cold” drug or cell line refers to those that were not included in the training set, meaning the model has not seen these drugs or cell lines during training. Conversely, a “warm” drug or cell line refers to those that were included in the training set. In the scenario of warm, the samples of validation set and the test set contain drugs or cell lines that has already existed in the training set. This particular setting is strategically employed for tasks such as phenotypic drug repurposing and drug mechanism discovery. In the “cold cell” and “cold drug” scenarios, the test set consists of cells and drugs that are entirely absent from the training set, respectively. These scenarios are designed to evaluate the models' transfer capabilities in the context of phenotypic screening. The “cold both” scenario entails both drugs and cells in the test set being completely unseen in the training set. Although this represents a formidable challenge, it stands as the most crucial scenario for precision medicine applications.

### Experimental Setup for Drug Target Prediction

First, we curated a target prioritization dataset for benchmarking methods. This process involved the manual collection of bioactivity data from PubChem for 305 drugs in the drug response dataset. Following this data collection, we employed a filtration process to obtain the active drug‐target pairs. Additionally, we transformed the protein identifiers into their corresponding Uniprot IDs for consistency. Consequently, the pair consisting of the drug and the 16430 human proteins in BioHG removing the positive samples are regarded as negatives. In summary, we amassed a substantial count of 9263 positive DTI pairs alongside a staggering 5003229 negative DTI pairs. For DTI prediction methods training, we harnessed the DTIs within the BioHG as positive samples in the training dataset. Concurrently, we employed the sampled negative DTIs for predicting drug responses in the above section as negative samples in the training dataset.

Three DTI prediction methods are employed as baseline models: MLP, TransformerCPI, and DrugBAN. MLP is a powerful and easily assessed method that finds applicability across a wide spectrum of drug discovery tasks. MLP is a two‐layer neural network that uses a 2048‐dimensional input vector formed by concatenating two components: the 1024‐dimensional Morgan Fingerprint for drug structures and the 1024‐dimensional ProteinBERT^[^ ^52^ ^]^ embeddings for protein features. TransformerCPI is a sequence‐based compound‐protein interaction (CPI) prediction method that leverages the Transformer's ability to model complex relationships and dependencies in CPI data. Typically, the model takes the SMILES sequence of drugs and protein sequences as input, encoding them into meaningful representations. Through a series of self‐attention mechanisms and feedforward layers, TransformerCPI learns to capture intricate patterns and associations between compounds and their target proteins. DrugBAN is an advanced DTI prediction method that works on drug molecular graphs and target protein sequences to perform prediction, with conditional domain adversarial learning to align learned interaction representations across different distributions for better generalization on novel DTIs. Additionally, we constructed a version of KGDRP that excludes drug response data (KGDRP_nop). In this version, the HGNN integrates a DTI predictor and a biological process predictor, operating on a heterogeneous graph constructed from DTI, protein‐pathway, and protein‐GO data.

Regarding the Mechanism of Action (MoA) analysis, we utilize Gene Ontology (GO) terms and pathway analysis to elucidate the biological context of protein targets. In this study, we quantified the occurrence frequency of the top 10 successfully validated targets' GO terms. These top 10 GO terms serve as proxies, succinctly encapsulating the potential biological functions of the protein targets. Pathway analysis, on the other hand, follows a distinct approach. The ranking of pathways associated with the top 10 protein targets relies on the scores provided by the biological process predictor within KGDRP. This methodology introduces a nuanced dimension to pathway prioritization, reflecting the biological processes relevant to our protein targets.

### Transfer Ability Assessment from High‐Throughput Screens to Pre‐Clinical Drug Screening

Cancer cell lines have been extensively utilized for drug development; however, they have demonstrated success in predicting clinical responses in only a limited number of cases. Therefore, in this section, the predictive performance of drug response was assessed when transitioning from high‐throughput screens to pre‐clinical drug screening. PDTX have emerged as invaluable tools in cancer research, serving as a bridge between traditional pre‐clinical models and clinical applications. In this study, the transferability of these models was evaluated using breast PDTXs sourced from Project Biobank1.^[^ ^22^ ^]^ Specifically, the expression profiles of the 20 PDTXs and the corresponding 1636 drug response data points were utilized for dataset construction. The origin data can be accessed at https://figshare.com/s/4a3f6bc543e5ba85834c. To obtain the structural information of drugs, SMILES data for drugs was manually collected from PubChem. Data points lacking drug SMILES were filtered out. Consequently, the external test dataset comprised 1595 drug response data points, encompassing 20 PDTX breast cancer models and 101 drugs.

Subsequently, the PDTX expression data was processed for BioHG integration. The initial step involved transforming the distribution of expression values into a standardized z‐score distribution to maintain consistency with GDSC data. Next, the gene symbols were converted to Uniprot IDs to facilitate data linkage within the BioHG, and association data between RNA and PDTX were utilized to update the BioHG. For the task of drug response prediction, formulated as a regression problem, the IC_50_ value was employed as the label. Given the wide range spanned by IC_50_ values, a logarithmic transformation (Log_10_) was applied, with a lower limit threshold set at ‐10 and an upper limit threshold set at 10. Due to the inherent disparities in bioactivity measurements across different laboratories, the evaluation metrics in this section were restricted to PCC and SCC. Two baseline methods were utilized for comparative analysis. The first method, DeepTTA, represents drug response data with minimal reliance on drug and cell line information. The second method involves a modified version of KGDRP, which omits BioHG data from its architecture. All three models were trained on the entire GDSC dataset using the hyperparameters for both scenarios. In the context of similarity analysis, cosine similarity was employed to calculate the PDTX model similarities based on the embeddings generated by KGDRP. To facilitate visualization, similarity scores between a single PDTX model and others were rescaled into the range of 0 to 1.

### Zero‐Shot Evaluation by Drug Repurposing for COVID‐19

Utilizing the SARS‐CoV‐2 expression data from GSE147507, we integrated the cell expression profiling of SARS‐CoV‐2 into the BioHG.^[^ ^34^ ^]^ To achieve this, the features of cells were limited to the common genes shared between the GDSC dataset and GSE147507, scaling both within the range of −1 to 1. Consequently, the well‐trained model provided representations for entities such as drugs, proteins, and cells. Leveraging these representations, we employed MLP to train a drug response predictor on the GDSC dataset. Notably, we framed it as a classification problem, modifying the ln *IC*_50_ by applying a threshold of −2, as suggested by Chang et al.^[^ ^53^ ^]^ Subsequently, we repurposed 7070 drugs existing in BioHG using the drug response predictor against four lung samples, encompassing two healthy and two SARS‐CoV‐2‐infected samples. The screening approach aimed to pinpoint drugs with elevated scores for SARS‐CoV‐2‐infected samples and diminished scores for healthy samples, employing a threshold set at 0.5. Moreover, we extended our drug repurposing efforts by employing two baseline models: MLP and DeepTTA. The MLP processes concentrated features derived from scaled gene expressions of cells and the molecular fingerprint of drugs, while DeepTTA takes in the concentrated feature representation of scaled gene expressions of cells and the SMILES of drugs. In the model training phase for cell‐target‐drug analysis, KGDRP incorporates an MLP‐based cell classification predictor. This classifier categorizes the 78 cells into infected or non‐infected states. Following this classification, the significance of each gene concerning COVID‐19 is assessed utilizing the integrated gradients method. Integrated gradients serve as an attribution method, providing insights into the importance of input features for a model's predictions. In this context, the prediction model *F*(*x*) employed for explanation is KGDRP, with the reference input *x*′ representing a state where all features are set to zero. The integrated gradients can be expressed as follows:
(10)

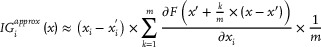

where *m* defines the number of interpolation steps. The rationale behind integrated gradients is to attribute the model's output to expression profile of cell lines, providing insights into their contributions and aiding in model interpretability. The network visualization and analysis are conducted using NetworkX.^[^ ^54^ ^]^ Specifically, we use betweenness centrality to characterize the importance of each gene in elucidating the relationship of screened drugs and cell line‐related gene. Betweenness centrality measures a node's influence on information flow in a graph by assessing its occurrence on shortest paths between other nodes, aiding tasks like finding critical proteins in biological networks.^[^ ^55^ ^]^ The betweenness centrality of a node *v* is given by the expression:

(11) $$g\left(v\right)=\sum_{s\neq v\neq t}\frac{\sigma_{st}\left(v\right)}{\sigma_{st}}$$

where σ_*st*_ is the total number of shortest paths from node *s* to node *t* and σ_*st*_(*v*) is the number of those paths that pass through *v* (not where *v* is an end point).

## Conflict of Interest

The authors declare no conflict of interest.

## Author Contributions

Q.Y., C.Y.H., S.B.H., and T.J.H. performed conceptualization: Q.Y. and C.Y.H. performed methodology: Q.Y., L.L.J., and Y.F.D. performed data process: Q.Y., Y.D.Z., and H.T.Z. performed experiments: C.Y.H., S.B.H., T.J.H., and J.M.C. performed supervision: Q.Y. wrote original draft: Q.Y., C.Y.H., T.J.H., S.B.H., J.M.C., Y.K., P.C.P. wrote, reviewed, and edited.

## Supporting information



Supporting Information

## Data Availability

The data that support the findings of this study are available from the corresponding author upon reasonable request.
