# Supplementary material for: A Knowledge‐Guided Graph Learning Approach Bridging Phenotype‐ and Target‐Based Drug Discovery
Source: Adv Sci (Weinh). 2025 Mar 6;12(16):2412402. doi: 10.1002/advs.202412402 (PMC12021103; doi:10.1002/advs.202412402)
Supplement: Supplementary file 1 — Supporting Information [file ADVS-12-2412402-s001.docx]

**Supplementary Materials for**

**A Knowledge-Guided Graph Learning Approach Bridging Phenotype- and Target-Based Drug Discovery**

Qing Ye *et al.*

*Chang-Yu Hsieh. Email: kimhsieh@zju.edu.cn

*Tingjun Hou. tingjunhou@zju.edu.cn

*Shibo He. s18he@zju.edu.cn

**This PDF file includes:**

Figures S1 to S2

Tables S1 to S8

**Fig. S1.** **Distribution of Drug and Cell Line Similarity Across Training, Validation, and Test Sets in 20-Fold Cross-Validation.** Drug similarity calculated using molecular fingerprint by cosine similarity. Cell line similarity calculated using 978 gene expression profiles.


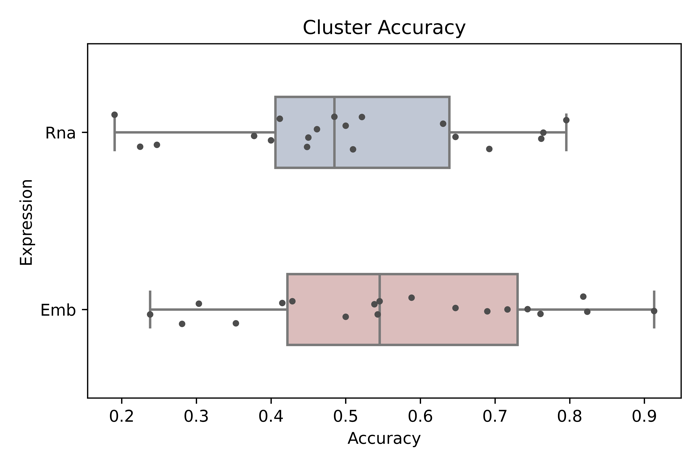
Fig. S2. The distribution of cluster accuracy.

Table S1. The number of the nodes and edges in the BioHG.

| Node Type | Node Number | Edge type | Edge Number | Data Source |
| --- | --- | --- | --- | --- |
| Drug | 7070 | Drug-Target Interaction | 28033 | DrugBank |
| Protein | 117841 | Protein-Protein Interaction | 234454 | BioKG |
| Pathway | 21178 | Pathway-Protein Association | 807539 | Reactome |
| Gene Ontology | 24210 | Protein-Gene Ontology Association | 891800 | UniProt |
| Cell Line | 804 | Protein-Cell line Association | 392554 | GDSC |
| Total | 171103 | Total | 2354380 | / |

Table S2. Performance comparison for drug response prediction between different BioHG

| **Metrics** | **Scenarios** | **KGDRP_BioKG** | **KGDRP_BioHG** |
| --- | --- | --- | --- |
| Num of Nodes | / | 218,787 | 146,893 |
| Num of Edges | / | 2,214,871 | 1,462,580 |
| Num of types of nodes |  | 5 | 4 |
| Num of types of edges | / | 13 | 4 |
| RMSE | Warm | 1.057 ± 0.009 | **1.019± 0.008** |
|  | Cold Cell | 1.376 ± 0.036 | **1.323± 0.032** |
|  | Cold Drug | 2.485 ± 0.279 | **2.288 ± 0.234** |
|  | Cold Both | 2.497 ± 0.255 | **2.174 ± 0.223** |
| Pearson | Warm | 0.919 ± 0.001 | **0.925 ± 0.001** |
|  | Cold Cell | 0.857 ± 0.008 | **0.868 ± 0.006** |
|  | Cold Drug | 0.438 ± 0.149 | **0.556 ± 0.115** |
|  | Cold Both | 0.410 ± 0.085 | **0.543 ± 0.075** |
| Spearman | Warm | 0.892 ± 0.001 | **0.901 ± 0.002** |
|  | Cold Cell | 0.809 ± 0.010 | **0.825 ± 0.008** |
|  | Cold Drug | 0.389 ± 0.128 | **0.516 ± 0.113** |
|  | Cold Both | 0.356 ± 0.069 | **0.496 ± 0.085** |

Table S3. Performance comparison on the pre-clinical dataset

| PTDX ID | Pearson correlation | | | Spearman correlation | | |
| --- | --- | --- | --- | --- | --- | --- |
|  | DeepTTA | KGDRP (no KG) | KGDRP | DeepTTA | KGDRP (no KG) | KGDRP |
| HCI001 | 0.040 | 0.176 | 0.207 | 0.154 | 0.179 | 0.261 |
| HCI002 | 0.178 | 0.318 | 0.329 | 0.194 | 0.256 | 0.297 |
| HCI009 | -0.008 | 0.177 | 0.184 | 0.109 | 0.160 | 0.206 |
| HCI010 | 0.433 | 0.541 | 0.549 | 0.429 | 0.502 | 0.556 |
| STG139 | 0.108 | 0.335 | 0.363 | 0.235 | 0.403 | 0.471 |
| STG139M | 0.184 | 0.364 | 0.388 | 0.323 | 0.409 | 0.489 |
| STG143 | 0.154 | 0.174 | 0.185 | 0.250 | 0.206 | 0.238 |
| STG195 | 0.396 | 0.495 | 0.526 | 0.393 | 0.487 | 0.549 |
| STG201 | 0.169 | 0.318 | 0.351 | 0.168 | 0.264 | 0.351 |
| STG282 | 0.463 | 0.452 | 0.456 | 0.452 | 0.448 | 0.482 |
| STG316 | 0.318 | 0.389 | 0.407 | 0.365 | 0.363 | 0.436 |
| STG335 | 0.337 | 0.416 | 0.414 | 0.316 | 0.407 | 0.448 |
| VHIO169 | 0.310 | 0.399 | 0.431 | 0.326 | 0.338 | 0.406 |
| VHIO179 | 0.198 | 0.221 | 0.245 | 0.291 | 0.272 | 0.341 |
| VHIO244 | 0.358 | 0.273 | 0.316 | 0.310 | 0.226 | 0.294 |
| HCI005 | 0.077 | 0.143 | 0.183 | 0.139 | 0.158 | 0.281 |
| HCI008 | 0.304 | 0.285 | 0.241 | 0.415 | 0.378 | 0.349 |
| HCI011 | 0.366 | 0.248 | 0.216 | 0.421 | 0.226 | 0.217 |
| IC007 | 0.025 | 0.019 | 0.158 | 0.035 | 0.036 | 0.180 |
| VHIO098 | 0.322 | 0.321 | 0.264 | 0.329 | 0.300 | 0.287 |
| **Mean** | **0.237** | **0.303** | **0.329** | **0.283** | **0.301** | **0.364** |
| **Std** | **0.142** | **0.129** | **0.117** | **0.118** | **0.124** | **0.115** |

Table S4. The metrics of Hits@50 and Hits@100 of lestaurtinib and PF562271.

| Drug | Metrics | MLP | TransformerCPI | DrugBAN | KGDRP_nop | KGDRP |
| --- | --- | --- | --- | --- | --- | --- |
| lestaurtinib | Hits@50 | 0 | 3 | 2 | 24 | **47** |
|  | Hits@100 | 0 | 6 | 6 | 45 | **84** |
| PF562271 | Hits@50 | 0 | 0 | 0 | 2 | **6** |
|  | Hits@100 | 0 | 1 | 0 | 5 | **10** |

Table S5. The ranking results of the top 10 validated proteins for drug lestaurtinib.

| **Candidate proteins** | **MLP** | **TransformerCPI** | **DrugBAN** | **KGDRP_nodrp** | **KGDRP** |
| --- | --- | --- | --- | --- | --- |
| Q92918 | 10484 | 2734 | 80 | 44 | 1 |
| Q8NEV4 | 8045 | 3352 | 7992 | 673 | 2 |
| Q86YV6 | 13514 | 148 | 10106 | 602 | 3 |
| Q8N568 | 14691 | 8893 | 7153 | 344 | 4 |
| Q13237 | 3877 | 11247 | 10149 | 1445 | 5 |
| Q9P2K8 | 13588 | 10982 | 4978 | 38 | 6 |
| P46734 | 8648 | 8037 | 8998 | 162 | 7 |
| O00444 | 15675 | 695 | 5887 | 154 | 8 |
| O95835 | 13375 | 8552 | 12008 | 14 | 9 |
| P54762 | 11141 | 965 | 6423 | 3 | 10 |

Table S6. The ranking results of the top 10 validated proteins for drug PF562271.

| **Candidate proteins** | **MLP** | **TransformerCPI** | **DrugBAN** | **KGDRP_nodrp** | **KGDRP** |
| --- | --- | --- | --- | --- | --- |
| Q92918 | 8287 | 784 | 444 | 142 | 1 |
| Q7KZI7 | 14927 | 5964 | 404 | 1569 | 13 |
| Q9NSY1 | 13000 | 1911 | 2027 | 476 | 21 |
| Q96L34 | 14335 | 1880 | 4736 | 648 | 40 |
| P16591 | 7272 | 2427 | 7389 | 293 | 42 |
| Q16512 | 7501 | 9212 | 272 | 61 | 50 |
| P52564 | 14921 | 2177 | 14227 | 678 | 66 |
| P27448 | 15916 | 1901 | 5609 | 1733 | 70 |
| P07947 | 15896 | 276 | 6783 | 7 | 76 |
| Q16584 | 12204 | 1248 | 5382 | 93 | 89 |

Table S7. Successfully validated repurposed drugs by KGDRP.

| **Drug ID** | **Drug Name** | **Relation with COVID** | **Source** |
| --- | --- | --- | --- |
| DB04977 | Plitidepsin | Plitidepsin has shown promise in treating COVID-19. It demonstrated favorable long-term safety in hospitalized adult patients. | KGDRP / MLP |
| DB13527 | Proglumetacin | proglumetacin (tested twice independently, with AC50 of 8.9 μM and 12.5 μM) | KGDRP |
| DB00199 | Erythromycin | Erythromycin, retapamulin, pyridoxine, folic acid, and ivermectin inhibit cytopathic effect, papain-like protease, and MPRO enzymes of SARS-CoV-2. | KGDRP |
| DB03880 | Batimastat | It has been found that batimastat-sensitive metalloproteases efficiently activate SARS-CoV-2 spike-mediated cell-cell fusion. | KGDRP |
| DB00256 | Lymecycline | In a computational study, it was identified as having the potential to bind to various targets associated with SARS-CoV-2, including Mpro, spike protein, RdRp, and furin. | KGDRP |
| DB01256 | Retapamulin | Erythromycin, retapamulin, pyridoxine, folic acid, and ivermectin inhibit cytopathic effect, papain-like protease, and MPRO enzymes of SARS-CoV-2 | KGDRP / MLP |
| DB00522 | Bentiromide | bentiromide has been identified as a potential papain-like protease (PLpro) inhibitor, a crucial enzyme for the virus's replication. | KGDRP |
| DB01601 | Lopinavir | Remdesivir, lopinavir, emetine, and homoharringtonine inhibit SARS-CoV-2 replication in vitro. | KGDRP / DeepTTA / MLP |
| DB04786 | Suramin | Suramin demonstrate its efficacy in hampering viral genome packaging, reducing the inhibition of type I interferon, and providing a potential treatment for COVID-19 patients, especially those in intensive care | KGDRP |
| DB02010 | Staurosporine | A cell-impermeable staurosporine analog targets extracellular kinases, inhibiting both HSV and SARS-CoV-2. | KGDRP |
| DB00615 | Rifabutin | Rifabutin have a potential inhibitory interaction with RdRp of SARS-CoV-2 and could be effective drugs for COVID-19. | DeepTTA |
| DB11617 | Aclarubicin | Aclarubicin and ecteinascidin-770 inhibit SARS-CoV-2 RdRp-mediated gene expression | DeepTTA |
| DB00200 | Hydroxocobalamin | Hydroxocobalamin, a form of Vitamin B12, exhibits potent antiviral activity against severe viral infections, including COVID-19. | DeepTTA |
| DB00602 | Ivermectin | Ivermectin has been shown to inhibit the replication of SARS-CoV-2 in cell cultures. | MLP |
| DB04297 | Trichostatin A | Trichostatin A binds to the surface groove of SARS-CoV-2 M-protein with high stability, suggesting a potential inhibitory effect on the virus. | MLP |
| DB11779 | Danoprevir | Danoprevir has been evaluated in an open-label, single arm clinical trial in combination with ritonavir for treating COVID-19 | MLP |

Table S8. Successfully validated COVID-19 related genes.

| **Gene** | **Association with COVID-19** |
| --- | --- |
| NFKB2 | Many immune-related signaling pathways (Fc-epsilon, NF-κB/NFKB2, and C-type lectin receptor) could be activated in the lungs of COVID-19 patients. |
| ARID4B | ARID4B (HDAC complex) are associated with therapeutics for SARS/COVID-19, inhibition of HDAC in COVID-19 treatment seems to occur by reducing ACE2, a receptor for SARS-CoV-1/2 entry into cells. |
| RELB | The nuclear translocation of RELB, a component of the NF-κB pathway, is increased in diseased states, suggesting its involvement in the host response to infections like SARS-CoV-2. |
| TNIP1 | TNIP1 is a hub protein associated with autoimmune diseases and showed differential expression in the transcriptomic profile of SARS-CoV-2 infected patients. |
| LAP3 | Comprehensive proteomics revealed LAP3 changes in COVID-19 plasma, indicating its involvement in host perturbations by SARS-CoV-2. |
| SOCS2 | An upregulation of SOCS2 (suppressor of cytokine signaling 2 implies a dysregulated cytokine/chemokine response, which may be the correlate of the cytokine storm observed in severe COVID-19 |
| BHLHE40 | BHLHE40, also known as DEC1, plays a role in the immune response to SARS-CoV-2. It controls cytokine production by T cells and is essential for immune regulation in severely ill COVID-19 patients |
| JUN | Persisting immune activation involving AP-1/p38MAPK was a specific feature of COVID-19. |

Table S9. Hyperparameters tuning in the KGDRP.

| **Hyperparameter** | **Description** | **Type** | **Search Space** |
| --- | --- | --- | --- |
| hid_feats | Number of hidden features | Integer | 256 to 1000 |
| aggregator_type | Type of aggregator | Categorical | ['gcn', 'mean'] |
| feat_drop | Dropout rate for features | Categorical | [0, 0.1, 0.2, 0.3] |
| patience | Early stopping patience | Integer | 8 to 20 |
| sample_pc_size | Sample size of PC triples | Integer | 1000 to 100000 |
| sample_dc_size | Sample size of DC triples | Integer | 1000 to 100000 |
| dc_mlti | Negative/Positive ratio for DC triples | Categorical | [0.1, 0.5, 1, 2] |
| pc_mlti | Negative/Positive ratio of PC triples | Categorical | [0.1, 0.5, 1, 2] |
| sample_propath_size | Sample size of Protein-Pathway pairs | Integer | 1000 to 10000 |
| sample_dpi_size | Sample Size of DPI | Integer | 1000 to 10000 |
| propath_mlti | Negative/Positive ratio of Protein-Pathway pairs | Categorical | [0.1, 0.5, 1, 2] |
| dpi_mlti | Negative/Positive ratio of DPIs | Categorical | [0.1, 0.5, 1, 2] |
| w_pc | Weight for PC triples | Categorical | [0.001, 0.1, 1] |
| w_dc | Weight for DC triples | Categorical | [0.001, 0.1, 1] |
| w_pro_path | Weight for Protein-Pathway | Categorical | [0.001, 0.1, 1] |
| w_dpi | Weight for DPI | Categorical | [0.001, 0.1, 1] |

Table S10. Hyperparameter configurations for KGDRP across four scenarios.

| **Hyperparameter** | **Warm** | **Cold Cell** | **Cold Drug** | **Cold Both** |
| --- | --- | --- | --- | --- |
| hid_feats | 691 | 784 | 645 | 364 |
| aggregator_type | 'mean' | 'mean' | 'mean' | 'mean' |
| feat_drop | 0.1 | 0.1 | 0.1 | 0 |
| patience | 15 | 9 | 3 | 3 |
| sample_pc_size | 17365 | 9626 | 39547 | 3541 |
| sample_dc_size | 78768 | 46176 | 10323 | 31970 |
| dc_mlti | 1 | 1 | 1 | 1 |
| pc_mlti | 0.1 | 0.1 | 1 | 0.5 |
| sample_propath_size | 4477 | 1210 | 4196 | 1316 |
| sample_dpi_size | 2216 | 2898 | 8529 | 6729 |
| propath_mlti | 2 | 2 | 1 | 2 |
| dpi_mlti | 1 | 0.5 | 2 | 1 |
| w_pc | 0.001 | 0.001 | 1 | 1 |
| w_dc | 0.1 | 0.1 | 0.001 | 1 |
| w_pro_path | 1 | 0.1 | 1 | 1 |
| w_dpi | 0.001 | 1 | 0.001 | 0.1 |
